# Correlated Wave Functions for Electron–Positron Interactions in Atoms and Molecules

**DOI:** 10.1021/acs.jctc.1c01193

**Published:** 2022-03-25

**Authors:** Jorge Alfonso Charry Martinez, Matteo Barborini, Alexandre Tkatchenko

**Affiliations:** Department of Physics and Materials Science, University of Luxembourg, L-1511, Luxembourg City, Luxembourg

## Abstract

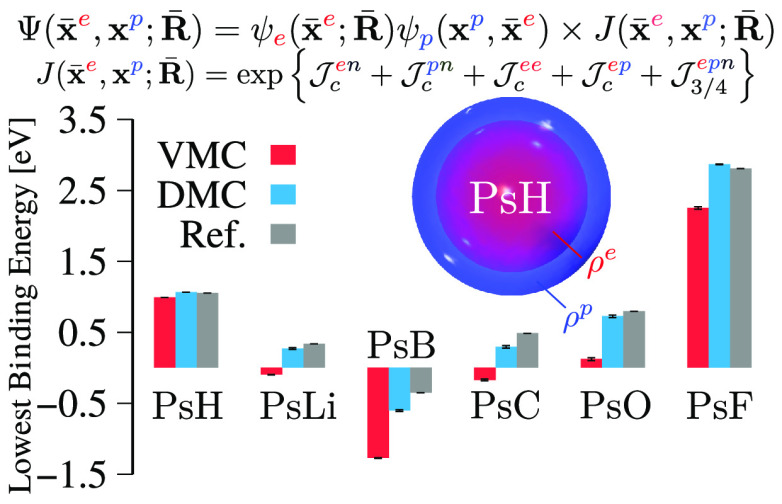

The positron, as the antiparticle of the electron, can form metastable states with atoms and molecules before its annihilation with an electron. Such metastable matter–positron complexes are stabilized by a variety of mechanisms, which can have both covalent and noncovalent character. Specifically, electron–positron binding often involves strong many-body correlation effects, posing a substantial challenge for quantum-chemical methods based on atomic orbitals. Here we propose an accurate, efficient, and transferable variational ansatz based on a combination of electron–positron geminal orbitals and a Jastrow factor that explicitly includes the electron–positron correlations in the field of the nuclei, which are optimized at the level of variational Monte Carlo (VMC). We apply this approach in combination with diffusion Monte Carlo (DMC) to calculate binding energies for a positron *e*^+^ and a positronium Ps (the pseudoatomic electron–positron pair), bound to a set of atomic systems (H^–^, Li^+^, Li, Li^–^, Be^+^, Be, B^–^, C^–^, O^–^ and F^–^). For PsB, PsC, PsO, and PsF, our VMC and DMC total energies are lower than that from previous calculations; hence, we redefine the state of the art for these systems. To assess our approach for molecules, we study the potential-energy surfaces (PES) of two hydrogen anions H^–^ mediated by a positron (*e*^+^H_2_^2–^), for which we calculate accurate spectroscopic properties by using a dense interpolation of the PES. We demonstrate the reliability and transferability of our correlated wave functions for electron–positron interactions with respect to state-of-the-art calculations reported in the literature.

## 1 Introduction

The positron,^1^ as the antimatter analogue of the electron, has many useful spectroscopic applications in chemistry, biology, and materials science^2−4^ based on the detection and analysis of γ rays produced during the electron–positron annihilation process. Furthermore, the techniques to accumulate and manipulate positrons^5^ and positronium atoms^6^ at low energies have greatly advanced, allowing significant breakthroughs, such as the production of dipositronium (Ps_2_),^7^ the development of positronium gamma-ray lasers,^8^ and the production of long-lived positronium beams to study gravitational interactions.^9^

Positrons can also interact with atoms and molecules before the onset of electron–positron annihilation (which happens on the time scale of 10^–9^ seconds). For example, it has been experimentally observed that positrons can form metastable bound states with atomic and molecular systems, and their positron–molecule binding energies have been measured within a resonant annihilation process, in which the positron binds to vibrationally excited molecules, apparently driven mainly by electrostatic and polarization interactions.^10−12^

These experimental findings have stimulated a wide range of theoretical studies,^12^ which have suggested various binding mechanisms, such as the formation of a positronic chemical bond between two otherwise repelling anions and a new series of energetically stable atoms^11,13,14^ and molecules^15−20^ bound by one positron. Furthermore, it has been shown that positrons can act as a chemical mediator by drastically changing energy profiles of proton-transfer reactions in amino acid compounds.^21^ Hence, positron binding could be envisioned as a useful mechanism for controlling chemical reactivity.

The prediction of positron binding to atoms and molecules requires robust theoretical methods that can handle both localized and delocalized positronic and electronic states, and at the same time have the capacity to accurately capture electron–positron correlation energies, which can be rather large.^11^ One can rely either on the hierarchy of quantum-chemical post-Hartree–Fock (HF) methods or quantum Monte Carlo (QMC) methods. The main disadvantage of post-HF methods lies in the use of single-particle atomic centered basis sets that are unable to properly describe the formation of bound states between electron–positron pairs. As a result, many of these methods including many-body perturbation theory (MBPT) and configuration interaction (CI) struggle to accurately describe positron–matter systems^22^ and have to rely on large basis set extrapolations and multireference approaches at an extreme computational cost, limiting their applicability to relatively small systems.^12,23,24^ The same limitations affect potentially highly accurate approaches such as explicitly correlated methods^25−27^ and the stochastic variational method (SVM),^28,29^ in which the dependence on interparticle distances is explicitly introduced in the wave function.

For these reasons, the most robust compromise is to study electron–positron systems through explicitly correlated wave functions optimized through QMC methods, as already done in previous studies.^30−36^ QMC methods are a family of stochastic integration techniques applied to compute physical observables over chosen trial wave functions that approximate the ground state. The main advantage of these approaches lies in the possibility to work with wave functions that are able to include explicit many-body interactions between the degrees of freedom in the system, greatly enhancing the accuracy and improving the convergence with respect to the basis set size. Computationally, QMC methods suffer from a large prefactor due to their stochastic nature; yet, for large systems, this is compensated by the intrinsic parallelism of the algorithms that can optimally exploit the rapid computational advancements toward exascale high-performing computing (HPC) facilities.^37^ Thus, the main challenge in QMC remains the definition of a variational ansatz for the wave function, which is able to represent the basic properties of the system examined, while still being generalizable and scalable with the number of particles.

For positron–matter systems the ansatz should also include, besides the standard electronic correlation effects and cusp conditions, the electron–positron correlation interactions, satisfying the nucleus–positron, electron–positron, and eventually positron–positron cusp conditions, together with the correct asymptotic behaviors as a function of the interparticle distances.^38^

To fulfill these requirements, several ansätze have been proposed in the literature, essentially based on three different approaches: The first is to consider the electron–positron interaction directly in the determinantal part of the wave function;^30,38−41^ the second is to include this correlation effect through a two-body Jastrow factor constructing the single particle positronic orbital as a linear combination of an atomic centered basis set,^33−35,42^ as usually done for the electrons; the third is to include the correlation effects through an electron–positron orbital that explicitly depends on the two particle distances and that multiplies the purely electronic wave function.^27,30,43^

The first approach, although the most accurate, is also the most complicated to generalize to large molecular systems. Thus, in this work, we compare the second and third types of wave function, discussing the crucial differences in recovering the electron–positron correlation, and introducing a novel three-body Jastrow factor^44^ with the purpose of recovering the correlation between electrons and positrons in the field of the atomic nuclei, achieving a robust improvement in the description of the positron–matter interactions. We apply this novel wave function to study in a systematic way the binding energies of the positron and positronium with the first row atoms, anions, or cations (H^–^, Li^+^, Li, Li^–^, Be^+^, Be, B^–^, C^–^, O^–^, and F^–^). In particular for the largest positron–atom systems, PsB, PsC, PsO, and PsF, we show that our QMC total energies are lower than results available in the literature.

In addition, we study the potential-energy surface (PES) describing the bond formation of the repelling H^–^ anions mediated by one positron^15−20^ demonstrating the robustness of our approach that can be generalized to larger electron–positron systems, and reporting accurate estimations of its spectroscopic properties.

This article is organized as follows: In section 2 we briefly describe the VMC and DMC methods. In the section that follows we explicitly define how we construct the electron–positron wave function. Next, in section 4 we show the total energies and the positron binding energies for several positronic atomic and molecular systems. Finally, in section 5 we summarize the results and provide concluding remarks.

## 2 Quantum Monte Carlo

Quantum Monte Carlo (QMC) methods^37,45,46^ are a set of stochastic techniques used to integrate physical observables over a given quantum state. The most common method is variational Monte Carlo (VMC), which stochastically estimates the energy functional *E*[Ψ_*T*_(**x̅**)] = (∫Ψ_T_^*^(**x̅**)ĤΨ_*T*_(**x̅**) d**x̅**)/(∫|Ψ_*T*_(**x̅**)|^2^ d**x̅**) over a chosen trial state Ψ_*T*_(**x̅**), where Ĥ is the Fermionic Hamiltonian operator and **x̅** is the vector of Cartesian and spin coordinates of the *N*_*f*_ Fermions system.

To stochastically compute the energy functional, the integrand is rewritten as the product of two functions *E*[Ψ_*T*_(**x̅**)] = ∫*E*_*l*_(**x̅**)Π(**x̅**) d**x̅**, which correspond to the local energy *E*_*l*_(**x̅**) = HΨ̂_*T*_(**x̅**)/Ψ_*T*_(**x̅**) and to the probability density proportional to the square modulus of the trial wave function Π(**x̅**) ∝ |Ψ_*T*_(**x̅**)|^2^. By sampling through the Metropolis algorithm  Fermionic configurations **x̅**_*i*_ distributed according to Π(**x̅**), the value of the energy functional can be obtained as the mean value  over the local energies, with the associated error  that decreases as the square root of the number of samples, with  equal to the variance of the local energy.

Within this variational framework many different minimization procedures have been developed to optimize the trial wave function over a set of predefined parameters, obtaining the best possible estimation of the ground state eigenvalue and eigenstate,^47−52^ of which the most successful is probably the Linear Method.^53−55^ In this work we will use the Stochastic Reconfiguration (SR) introduced by Sorella in ref (56) and successfully applied to optimize atomic and molecular wave functions in ref (57).

To improve the treatment of quantum many-body effects and overcome the limitations of the variational wave function’s parametrization, here in addition we apply the diffusion Monte Carlo (DMC) method.^37,45,46^ DMC is a projection technique based on the wave function propagation in imaginary time that is able to converge to the ground state of a Fermionic system within the Fixed-Node (FN-DMC) approximation. The FN-DMC overcomes the sign problem of the standard DMC algorithm, by fixing the nodal surface of the projected wave function to that of the trial wave function, and relaxing its amplitudes. In this way one obtains the best estimation of the ground state for a particular nodal surface, recovering dynamical correlation between Fermions and obtaining a more accurate estimation of the corresponding observables. In our work, we use the recently implemented method from Zen et al.^58^ to reduce the dependency of the binding energy estimations on the time discretization.

The methods are implemented in QMeCha α.0.3.0,^59^ a QMC package published on Github.

## 3 Electron–Positron Wave Functions

The most general expression for many-electrons and a positron wave function Ψ(**x̅**^*e*^, **x**^*p*^; **R̅**) explicitly describes the many-body correlation effects between the 4*N*_*e*_ electronic Cartesian and spin coordinates **x̅**^*e*^ and the four positronic **x**^*p*^ coordinates in the field of the nuclei **R̅**.

A first approximation to this fully correlated state can be built by considering only the explicit correlation between particle pairs. The wave function is thus built as a symmetrized product (or a linear combination of symmetrized products) of two-particle functions, as proposed for example by Bressanini et al. in ref (39), describing the correlation between electron–electron, electron–positron, nucleus–electron, and nucleus–positron pairs. Clearly, this ansatz, although very accurate, is more computationally expensive when applied to large systems of many atoms and many positrons.

A way to further simplify the total wave function is that of decoupling it into a product
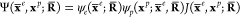
1of two Fermionic functions, an electronic one ψ_*e*_(**x̅**^*e*^; **R̅**) (such as a Slater determinant) and a positronic orbital ψ_*p*_(**x**^p^; **x̅**^*e*^, **R̅**), and a bosonic Jastrow factor that describes the correlation between the remaining particle pairs, eventually also including three or four body correlation effects, as we propose in this work.

Assuming that the electronic wave function ψ_*e*_(**x̅**^*e*^; **R̅**) describes the spin and angular symmetries of the electrons in the field of the nuclei, the general positronic function ψ_*p*_(**x**^*p*^; **x̅**^*e*^, **R̅**) will depend on both the nuclear and electronic coordinates, being symmetric for the exchange of any electronic coordinate.

In the literature the ψ_*p*_(**x**^*p*^; **x̅**^*e*^, **R̅**) function has been further simplified assuming it to be independent from **x̅**^*e*^^18,22,34,60,61^ or from **R̅**,^30^ the former chosen especially for computational reasons, since it is also simpler to implement and integrate with post HF methods.

In the following sections we discuss the three parts of the total wave function.

### 3.1 Electronic Wave Function

Because of the multiconfigurational nature of some of the electronic systems studied in this work, for example the Be atom and the Li^–^ anion, the electronic wave function is chosen to be the antisymmetrized geminal power (AGP)^62^ which corresponds to a more compact and constrained multideterminantal expansion.^63^ For a closed shell system the AGP is built as the determinant
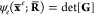
2of a *N*_*e*_^↑^ × *N*_*e*_^↓^ matrix **G**, the elements **G**_*ij*_ of which describe the coupling of electronic pairs in a singlet state 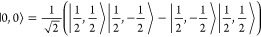
through the symmetric linear combination of products of two atomic orbitals modulated by the coupling coefficients λ_*qp*_:
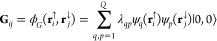
3For a spin polarized systems (*N*_*e*_^↑^ > *N*_*e*_^↓^) the geminal matrix can be generalized^64^ by adding *N*_*e*_^*u*^ = *N*_*e*_^↑^ – *N*_*e*_^↓^ columns, each with *N*_*e*_^↑^ elements, containing unpaired molecular orbitals
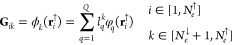
4occupied solely by the spin up electrons: In this way we reconstruct a square **G** matrix of *N*_*e*_^↑^ × *N*_*e*_^↑^ elements.

### 3.2 Positronic Wave Function

A very common approach in the literature^18,22,34,60,61^ assumes that the positronic wave function is independent of the electronic coordinates, and can be written as positronic molecular orbitals (PMO) which are a linear combination
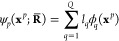
5of atomic orbitals ϕ_*q*_(**x**^*p*^), in which we have hidden the nuclear coordinates on which the orbitals are centered. This kind of approach is well suited when describing the positron’s interactions with atoms or anions, since its density is distributed spherically around the electronic charge. Yet, for molecules such an approach becomes deficient, since while the positron forms bound states with the electrons to which it is attracted, it does not form bound states with the atomic nuclei that repel it.

One way to solve this inconsistency is to construct the positron’s orbital through a positronic basis set^30,39^ explicitly describing the bound states between electron–positron pairs. As a matter of fact, it can be easily shown that the ground state of a system of one electron and one positron, that is, the positronium (Ps), can be exactly described by an exponential function of the electron–positron distance *r*^*ep*^ = |**x**^*e*^ – **x**^*p*^|:
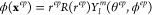
6where *R*(*r*^*ep*^) is a radial function normalized with respect to the distance *r*^*ep*^ and *Y*_*l*_^*m*^(θ^*ep*^,ϕ^*ep*^) is a real spherical harmonic (centered on the positron) that is used to introduce an angular momentum.^65^

Through this basis we can construct a positronic wave function for many electrons and one positron as the product 
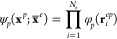
7of identical orbitals (so that the function is symmetric with respect to the exchange of the electronic coordinates), each dependent on the electron–positrons distance **r**_*i*_^*ep*^, thus referred to as electron–positron orbitals (EPO), that are defined as linear combinations
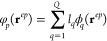
8of the newly defined positronic orbitals.

It can be shown that the scaling of the computational cost with respect to the number of electrons, of both the PMO and the EPO wave functions, is negligible with respect to that of the electronic determinant. It is in fact known that through the Sherman–Morrison formula^66^*N*_*e*_ consecutive updates of the electronic determinant require at most *N*_*e*_^3^ operations.

The PMO wave function, which is updated only when the positron’s coordinates are changed, requires at most *Q* multiplications (*Q* being the length of the atomic basis set which is proportional to *N*_*e*_), which is negligible with respect to the electronic determinant.

For the EPO, on the other hand, since the update of the wave function for the change of one electronic coordinate requires *Q* operations, *N*_*e*_ consecutive updates require *N*_*e*_*Q* multiplications, which is the same computational cost of the EPO update for the change of the positron’s coordinates. Thus, the full configuration update will cost 2*N*_*e*_*Q* operations for which *Q* is the length of the positronic basis defined in eq 6, which can also be set to one and that in any case is lower than the number of electrons. Again this means that the computational cost of the update of the EPO is negligible with respect to the update of the electronic determinant.

In this work we will compare the results obtained with both the PMO and the EPO based wave functions used in combination with a novel Jastrow factor to accurately recover the correlations between electron–positron pairs in the electrostatic field of the nuclei. This Jastrow factor is described in the next section.

### 3.3 Jastrow Factor

The bosonic Jastrow factor^44^ constructed in this work, that explicitly includes many body correlations in the QMC wave functions, is inspired by the general form introduced by Casula et al. in ref (62), as the linear combination of five terms
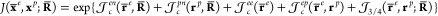
9which can be classified as one-body terms,  and , that are used to describe the Fermion-nucleus cusps conditions, pure homogeneous two-body terms,  and , that describe the pair correlations between electronic pairs and electron–positron pairs, and finally a many-body (or inhomogeneous) term  that is used to describe the Fermionic pair correlations in the field of the nuclei.

The one-body Jastrow factors are written as the sums
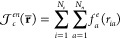
10
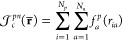
11of functions that only depend on the relative distances *r*_*ia*_ between the *i*th Fermion and the *a*th nucleus, and are used to reproduce the nuclear cusp condition.

The functions used to describe the nuclear cusp condition are different for electrons and the positron, due to the corresponding attractive and repulsive nature of the interactions. For this reason for the electron–nucleus cusp we use the short-range function^62^
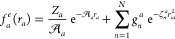
12while for the positron–nucleus cusp we use the long-range cusp^62^
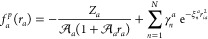
13where  is a factor depending on the nuclear charge and a remodulating variational parameter  that can depend on the atom: In all our calculations we fix this variational parameter simply to one. The sums that appear in the two equations are a linear combination of Gaussian functions centered on the corresponding atom that is modulated by a set of coefficients *g*_*n*_^*a*^ and γ_*n*_^*a*^ and by the corresponding exponents ζ_*n*_^*a*^ and ξ_*n*_^*a*^, that depend on the atom and are optimized.

The homogeneous two-body Jastrow factors that describe the correlation between electronic pairs and electron–positron pairs are also written as the sum of functions depending only on the distances between particle pairs
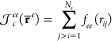
14
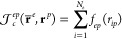
15The functions used to describe the Fermionic cusps conditions are different for the two types of particle pairs. For the repulsive electronic pairs we use the functions
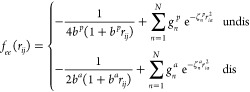
16respectively for distinguishable (antiparallel spin) electrons and undistinguishable ones (parallel spin). The variational parameters *b*^*p*^ and *b*^*a*^ are related to the cusp functions and are optimized independently.^67^ The additional linear combination of Gaussian type orbitals works as a remodulating factor depending on the set of coefficients *g*_*n*_^*p*^ and *g*_*n*_^*a*^ and exponents ζ_*n*_^*p*^ and ζ_*n*_^*a*^ that are optimized. For the attractive electron–positron cusp we use the short-range cusp function of the form:
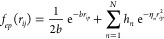
17where again *b*, the coefficients *h*_*n*_, and the exponents η_*n*_ are optimized variational parameters.

Finally, the last nonhomogeneous term in the Jastrow factor is a three/four body term, written as the linear combination of products of two atomic orbitals:
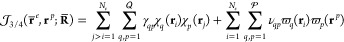
18in which the first group of elements describes the correlation of two electrons in the field of one or two nuclei and the second group of elements describes the correlation of the electron–positron pairs in the field of one or two nuclei. Here χ_*q*_(**r**) and ϖ_*q*_(**r**) are a set of atomic orbitals and γ_*qp*_ and ν_*qp*_ are a set of coefficients that are fully optimized.

This Jastrow term is an extension to the one introduced for pure electronic systems by Casula et al.^62^ and it is necessary to recover the dynamical correlation between Fermionic pairs, suppressing also nonphysical charge fluctuations.^68^ Since the Jastrow factor must be symmetric with respect to the exchange of all the electrons, the γ_*qp*_ parameters satisfy the condition γ_*qp*_ = γ_*pq*_.

Also for simplicity, in this work the two atomic basis sets are chosen to be identical, so that χ_*q*_(**r**) = ϖ_*q*_(**r**).

It is important to add that the presence of one positron does not change the original computational cost of the purely electronic dynamical Jastrow factor. In fact, by partially storing intermediate matrix–vector operations, it can be shown that the update of this Jastrow factor for the change of the positronic coordinate requires *QN*_*e*_ multiplications which is the same computational cost of the update of the dynamical Jastrow factor that describes correlation between electronic pairs. Thus, the update of the dynamical Jastrow factor for the change of all the Fermionic coordinates requires a number of multiplications that is proportional to *QN*_*e*_^2^ ∝ *N*_*e*_^3^, and comparable to the computational cost required for *N*_*e*_ consecutive updates of the electronic determinant and of its inverse matrix.

### 3.4 Computational Details

As discussed in the previous sections, to construct the electronic wave functions we have used the AGP with a basis set of contracted Gaussian type of orbitals (GTOs). In particular, for the H atoms we have used 3s1p Gaussian primitives contracted in the 1s1p orbitals, that is, (3s1p)/[1s1p]. For Li we have used a basis set of (5s4p1d)/[2s1p1d] contracted GTOs and for B, C, O, and F we have used a similar basis set of (6s4p1d)/[2s1p1d] contracted orbitals. These orbitals have been initialized before starting the full optimization by maximizing the overlap of the primitives’ linear combinations together with the one-body cusp function in eq 10, with the contracted orbitals from the Slater-type basis of Bunge et al.^69^ For the many-body Jastrow factor term described in eq 18, the χ_*q*_(**r**) and ϖ_*q*_(**r**) orbitals are assumed to be the same. In particular, for the H atoms we have used (3s2p) uncontracted GTOs, while for all the heavier atoms we have added one uncontracted d orbital, using the total basis of (3s2p1d) GTOs. Notice that during the optimization all the orbitals’ parameters are relaxed.

Also, the basis set used to construct the PMOs (eq 5) or the EPOs (eq 8) has been chosen to be simply made of contracted GTO functions with the same number of primitives [5s1p1d]/(1s1p1d). After the optimizations we have noticed that the higher angular momenta *p* and *d* were associated with very small coefficients, thus not contributing to the final wave function, as expected.

Finally, for all the cusp functions in eqs 10,11, 14, and 15, the number of additional Gaussian functions have been chosen to be equal to *N* = 5.

Regarding the DMC calculations, we have chosen to extrapolate to the continuum the energies obtained with approximately 2000 walkers and with d*t* = [0.015, 0.010, 0.005, 0.001].

## 4 Results and Discussion

### 4.1 Electron Affinities

As known from previous computational investigations^14^ of the neutral first row atoms, only Li and Be are known to bind with *e*^+^. On the other hand, *e*^+^ has been found to bind also with some of the anions such as H^–^, Li^–^, B^–^, C^–^, O^–^, and F^–^. To compute the energetic stability of these positronic systems with QMC and to study the behavior of the implemented wave functions, it is first important to verify the convergence of the electronic wave functions by computing the total energies and by evaluating the electron affinity (EA) and the ionization potential (IP) for the different atoms.

**Figure 1:**
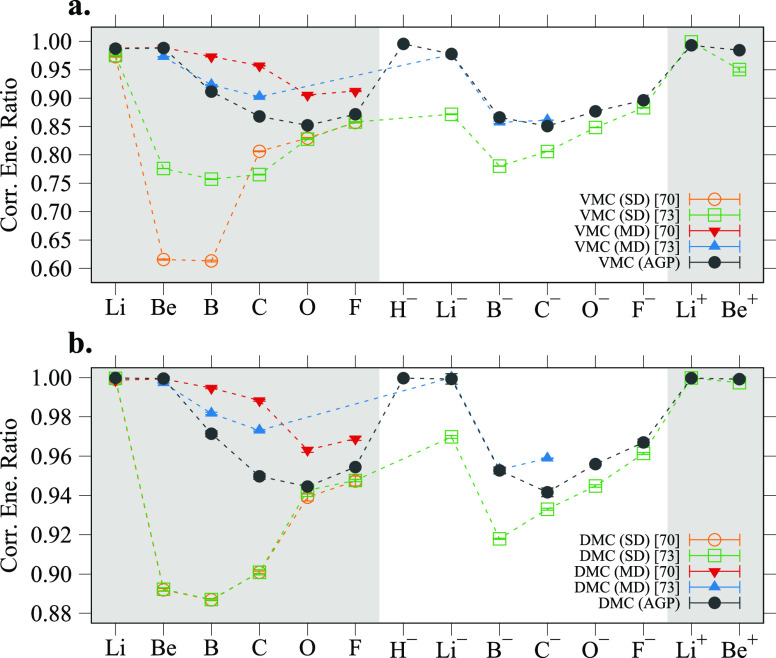
Correlation energy ratio defined as , between the *exact* reference^72^ and the correlation energies recovered by the AGP wave function, compared to the single-determinant (SD) and multideterminant (MD) results from ref (70) and ref (73) with VMC (panel a) and with DMC (panel b).

The values of the total energies, obtained using the AGP wave function with the VMC and DMC methods are reported in Tables S1 and S2 of the Supporting Information and compared to the accurate single-determinant (SD) and multideterminant (MD) calculations from refs (70 and 71). To simplify the comparison, in Figure 1 we show the correlation energy ratio recovered at the VMC level (panel a) and at the DMC level (panel b) defined as , where the *exact* reference corresponds to the most accurate nonrelativistic total energies of atoms obtained by Chakravorty et al.^72^ that estimated the correlation energy from experimental ionization potentials and complete active space (CAS) calculations. The differences in the energies in refs (70 and 71) are due to two factors. For the SD wave functions the authors used different basis sets and slightly different Jastrow factors. The differences within the two MD results occur because while Brown et al.^70^ converged the energies as a function of the number of configurations, including a number of determinants ranging from 83 for Li to 499 for Ne, Buendía et al.^71^ limited the number of configurations including only selective excitations involving 2p, 3s, 3p, and 3d orbitals.

Since the AGP wave function is a constrained MD expansion, it is able to include up to double excitations depending on the basis set. For this reason, the AGP energies of the Li and Be atoms, the wave functions of which require the inclusion of the nearly degenerate *p* orbitals, are comparable to the most accurate MD calculations from Brown et al.^70^ For the heavier atoms and anions, on the other hand, the AGP wave function greatly outperforms the single determinant, and is comparable with the results of Maldonado et al.^71,74^ that only include a limited number of configurations. Given the aforementioned reasons, it is evident that the AGP gives results that are in between the SD calculations and the MD ones, for all the atoms and ions taken into consideration, and converges toward the MD results for the lighter atoms, or for those atoms in which the used MD space was kept small.

Although these results seem to point toward rather accurate and converged estimations, some inconsistencies are observed when looking at energies differences such as EAs and IPs (Table 3S of the Supporting Information).

To the best of our knowledge a complete analysis of the EA and the IP for SD and MD wave functions with QMC methods has been done only by Maldonado, Buendía, and co-workers^74^ and for this reason from now on we will only discuss the comparison with their calculations.

Again to simplify the understanding of the results in Table 1 we report the relative error, defined as (*E*_calc_ – *E*_exp_)/*E*_exp_, between the calculated and experimental values in percentage. It can be seen that for H, Li, Be, O, and F the values computed with the AGP wave function have an accuracy within 5% of the experimental value with VMC and within less than 1% with DMC. Yet, for C and especially for B, the EA is greatly underestimated. This same discrepancy can also be observed for the MD wave function of ref (74) and is explained by the inconsistency between the multiconfigurational spaces of the neutral atom and its anion. This occurs because, especially for the AGP wave function, the addition of one electron removes the possibility to include double *p* excitations in the expansion of the anion, that are in fact present in the atom. As a consequence, the wave function of the atom is more accurate, and the energy difference between the two states is underestimated. This inconsistency is also at the root of what was observed with the AGP wave function for more complex molecules in ref (75). A way to correct this inconsistency and to verify its effect is to use for the boron atom a single SD wave function that seems to give more consistent results.

**Table 1:** Values of the Relative Errors (In Percentage), Defined as (*E*_calc_ – *E*_exp_)/*E*_exp_, for the Electron Affinities (EA) and Ionization Potentials (IP) of the Various Atoms, Obtained with the AGP Wave Function and Compared with the Single-Determinant (SD) and Multideterminant (MD) Results of ref (74)^a^

	VMC			DMC		
	SD^74^	MD^74^	AGP^b^	SD^74^	MD^74^	AGP^b^
EA_H_			–0.52(3)			0.1(8)
EA_Li_	–36.1(3)	–2.4(2)	–4.5(2)	–9.6(3)	0.2(2)	–0.2(8)
EA_B_	–15(1)	–108(3)	–82(2)	21.5(7)	–44(1)	–32(3)
EA_C_	2.1(3)	–22.4(2)	–14.8(6)	6.3(5)	–8.0(2)	–7(1)
EA_O_	–11.0(4)		–5(1)	–6(1)		–0.6(3)
EA_F_	–0.5(3)		0.2(2)	1.3(2)		1.3(5)
IP_Li_	–0.55(2)		–0.14(2)	–0.01(2)		0.01(2)
IP_Be_	–5.48(5)	–0.06(5)	–0.11(3)	–2.93(2)	–0.03(1)	–0.01(1)

a Values of the IPs and EAs are reported in Table 3S of the Supporting Information.

b This work.

The VMC and DMC energies computed with the SD wave function for B and B^–^ are reported in Tables 1S and 2S of the Supporting Information. Even if these SD energies are slightly lower than those reported in ref (74), they give values for the EA that are absolutely comparable, in fact we obtain 0.273(4) eV for VMC and 0.341(8) eV with DMC (Table 3S of the Supporting Information). This time while the VMC results are quite accurate, the DMC results appear to overestimate the EA by nearly 50% of its value. Thus, since the B atom remains the most complicated system that requires careful attention, in the next section, when computing its binding energy with the positron, we will use both the SD and AGP wave functions in order to compare the results.

### 4.2 Total Energies of Atomic–Positron System

Total energies for the positronic atoms are given in Table 2 at VMC and DMC levels employing the two positronic wave function ansätze PMO and EPO, while as electronic wave function we employed the AGP for all the atoms and the SD for the case of PsB, which will be discussed later in detail in this section. We compare our results with those previously obtained from VMC and DMC by Bressanini et al.^30,32^ with different types of wave functions. Additionally, we also report the most accurate values present in literature obtained with other methods such as the stochastic variational method (SVM),^77,80,81^ Multi-Reference Configuration Interaction (MRCI)^23,24,79^ and Hylleraas functions.^78^

**Table 2:** Nonrelativistic Total Energies of the Positron (*e*^+^) and the Positronium (Ps) Interacting with the Atomic Systems^a^

	e^+^Li(^2^S)	e^+^Be(^1^S)	PsH(^1^S)	PsLi(^1^S)	PsB(^3^S)	PsC(^4^S)	PsO(^2^P)	PsF(^1^S)
VMC SP^32^	–7.525 10(10)		–0.786 200(10)					
VMC MP^32^	–7.530 180(10)		–0.788 230(10)	–7.726 160(80)				
VMC^30^				–7.498 200(30)	–24.765(2)	–38.003 0(20)	–75.145 0(30)	–99.996 0(30)
VMC SD/PMO					–24.840 35(12)			
VMC SD/EPO					–24.840 97(13)			
VMC AGP/PMO	–7.523 02(11)	–14.657 7(33)	–0.785 600(37)	–7.722 950(85)	–24.845 635(81)	–38.067 27(37)	–75.280 46(81)	–100.021 99(67)
VMC AGP/EPO	–7.525 66(80)	–14.663 86(18)	–0.786 416(33)	–7.723 921(87)	–24.846 154(81)	–38.068 00(39)	–75.283 66(53)	–100.024 90(58)
DMC SP^32^	–7.531 650(80)		–0.789 160(30)					
DMC MP^32^	–7.532 290(20)		–0.789 150(40)	–7.739 529(60)				
DMC^30^				–7.737 600(40)	–24.875(1)	–38.095 90(60)	–75.317 70(50)	–100.071 90(80)
DMC SD/PMO					–24.873 89(26)			
DMC SD/EPO					–24.875 63(82)			
DMC AGP/PMO	–7.530 72(95)	–14.668 57(28)	–0.789 01(13)	–7.738 17(17)	–24.877 96(83)	–38.096 80(78)	–75.327 39(20)	–100.070 88(49)
DMC AGP/EPO	–7.530 94(23)	–14.669 31(36)	–0.789 119 1(31)	–7.738 04(41)	–24.878 19(37)	–38.097 95(57)	–75.329 69(63)	–100.074 35(15)
CI			–0.788 74(60)^b^		–24.830 56^c^	–38.053 62^c^	–75.281 27^c^	–100.001 817^d^
SVM	–7.532 323^28^	–14.669 042^28^	–0.789 196^76^	–7.740 208^77^				
Hylleras^78^			–0.789 196 714 7(42)					

a In parentheses we report the symmetry state of the electrons. All energies are reported in Hartree. AGP and SD are related to the electronic wave function: they indicate respectively the antisymmetrized geminal power and the Slater determinant. SP, i.e., single-pairing, corresponds to one antisymmetrized explicitly correlated pairing function from ref (32), while MP, i.e., multiple-pairing, corresponds to a linear combination of SP functions. For H the authors use a linear combination of 28 SP functions, while for Li they use 111.

b FCI extrapolation from ref (61).

c FCI limit with higher momentum corrections from ref (79).

d MRCI calculation from ref (24).

As expected, the EPO wave function provides lower energies because the dependency on the electron–positron distances are included explicitly into the wave function, differently from the PMO where these correlation effects are introduced only as remodulating factors through the Jastrow term. Nevertheless, both the EPO and PMO energies are comparable at the VMC level and are virtually identical at the DMC level for these atomic systems. This is because in atomic systems, where the positronic orbital is spherically symmetric and localized around the electronic charge, the atomic basis set expansion used in the PMO becomes a reasonable approximation. This is clearly not the case in molecules, as will be shown in the next section.

If we compare our results with those obtained by Bressanini et al. in ref (30) for the PsLi, PsB, PsC, PsO, and PsF systems, we can see that our VMC energies with both EPO and PMO wave functions are always lower. This is explained by two facts. First, in their VMC calculations the variational parameters of the electronic wave function were optimized for the neutral atoms and kept frozen in the positronic complex, thus optimizing the positronic orbital but preventing the distortion in the electronic density which is polarized by the positron. Second, the authors only use a two-body Jastrow factor, compared to our wave function that includes dynamical correlation effects of the electron–positron pairs in the field of the nucleus, through the dynamical Jastrow factor described in eq 18. Despite this, their DMC energies are comparable to ours, indicating that DMC is able to correct the electron–positron distribution, since most likely the positron does not drastically change the nodal surface of the electronic wave function.

The limitations of the wave function presented in ref (30) were fully discussed and improved by the same authors in a subsequent publication.^32^ In their work, Bressanini and co-workers proposed the use of a more accurate trial wave function, written as the antisymmetrized product of two-body pairing functions constructed between all the Fermionic or nuclear degrees of freedom in an Hylleraas-type ansatz. They construct the wave functions with only one of these antisymmetrized pairing functions, single-pairing (SP), and as linear combinations of many of these terms, multipairing (MP), applying them to compute the binding energies of the e^+^Li, PsH, and PsLi spherical systems at both the VMC and DMC levels.

Interestingly, by comparing the SP results with our AGP/EPO wave function we can see that at both the VMC and DMC levels there is an agreement for both the e^+^Li and PsH systems. This is because, the AGP/EPO includes the pair correlation between all particles in a combination of Jastrow and EPO function, and it can be thought of as an explicitly correlated single pairing function.

On the other hand, the MP results obtained by Bressanini and co-workers for the e^+^Li and PsLi systems are 5 mHa more accurate at the VMC level and around 1–2 mHa more accurate at the DMC level when compared to our AGP/EPO wave function. Thus, in order to improve our variational estimations, we would need to expand our variational ansatz in a combination of many AGP/EPO Fermionic terms, which is beyond the scope of this investigation.

Another proof of the accuracy of our approach can be found by examining the e^+^Be system, for which our DMC energy is exceptionally lower than the accurate value obtained with SVM.^77^ This is explained by the numerical difficulties the authors have faced to converge the ECG basis of 1275 functions. For this reason they decided to focus their efforts into improving the frozen core SVM polarization wave function which led to a bettering of the total energy prediction for e^+^Be, obtaining the value of −14.6705(1) Ha which is around 1 mHa lower than our DMC estimation. Finally, for the anionic positronic atoms (PsB, PsC, PsO, PsF) the only references present in the literature are the VMC and DMC results of Bressanini et al. from ref (30) and the extrapolated FCI energies of Saito in refs (23, 24, and 79). Regarding these last results, it is worth mentioning that their estimated total energies are 0.04 Ha higher than our DMC predictions on average, probably due to the frozen core approximation employed and to the fact that the CI expansion is written in terms of single-particle atomic basis sets.

In summary, for e^+^Li, e^+^Be, PsH, and PsLi, our best results obtained at the DMC AGP/EPO level are in good agreement with the highly accurate approaches based on explicitly correlated wave functions.^32^ Moreover, for PsB, PsC, PsO, and PsF, since our VMC and DMC values are always lower in energy with respect to the results previously published in the literature,^23,24,30,79^ we can conclude that they are the best energy references reported until now in the literature.

### 4.3 Positron Affinities and Positronium Binding Energies with Atoms

Having compared the total energies of our systems, assessing the quality of the overall results, we now study the binding energies of the positron, that is, positron affinity (PA), and of the positronium, that is, positronium binding energy (BE_Ps_), with atoms.

The PA is related to the direct binding of the positron to the electronic system, and it corresponds to the difference between the total energies of the system without and with the positron attached to it:
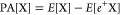
19similar to electron affinity. The BE_Ps_ is related to the separation of the electron/positron system into the electronic system with one less electron and positronium (with a total energy of −0.25 Ha); and is thus defined as the energy difference:
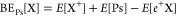
20

Considering the above definitions, when both channels give positive binding energies the positronic atom can be considered energetically stable, on the contrary, when even one of the two has a negative value this indicates that the system is predicted to dissociate according to that channel.

**Figure 2:**
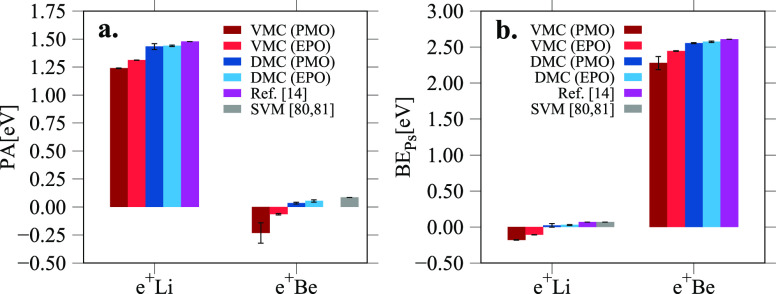
Positron affinities (PA) (panel a) and positronium binding energies (BE_Ps_) (panel b) of the *e*^+^X systems computed with the PMO and EPO wave functions using VMC and DMC methods. The results are compared to other references in the literature.

**Figure 3:**
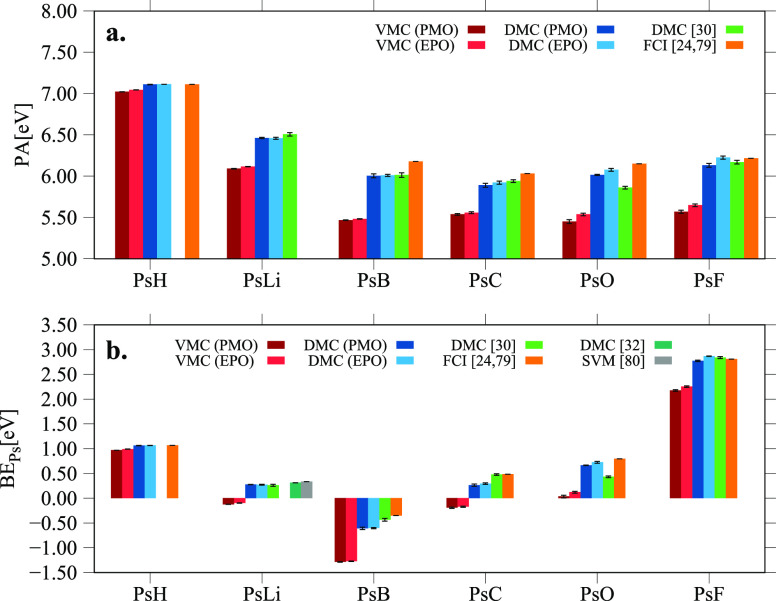
Positron affinities (PA) (panel a) and positronium binding energies (BE_Ps_) (panel b) of the PsX systems computed with the PMO and EPO wave functions using VMC and DMC methods. The results are compared to other references in the literature. For clarity purposes, the scale of the BE_Ps_ plot is intentionally chosen to cutoff the underestimated VMC values from ref (30)

In Table S4 of the Supporting Information we present the computed binding energies for all the atomic systems studied in this work using both dissociation channels in eq 19 and eq 21 at VMC and DMC levels employing the two positronic wave function ansätze PMO and EPO. For comparison we report in the same table also the best values present in the literature, including those obtained with the stochastic variational method (SVM),^80,81^ multireference configuration interaction (MRCI),^23,24,79^ Hylleras functions,^78^ the predicted values by Cheng et al.,^13^ the recommended values compiled by Harabati et al.,^14^ and those obtained through the VMC and DMC calculations done by Bressanini and co-workers.^30,32^ However, to simplify the discussion in the manuscript we present in Figures 2 and 3 the PA and BE_Ps_ data as bar plots.

Considering the performance obtained for the total energies, we assume that the best estimation, within those presented in this work, are the bindings obtained at the DMC level with the AGP/EPO wave function. On the basis of these calculations, we can in fact see that all the electron/positron systems are stable with respect to both the dissociation channels, except for B^–^ for which the BE_Ps_ is negative, predicting it to dissociate in Ps and the neutral B atom. These binding energies are comparable to the most accurate DMC,^30,32^ SVM,^80,81^ or MRCI^23,24,79^ calculations present in the literature for some of the systems.

Surprisingly, within the same QMC method, there are no appreciable differences between the binding energies obtained with the PMO or EPO wave functions. As discussed above, this probably occurs because the positronic orbital is spherically symmetric and centered around the atoms, making the atomic basis set of the PMO wave function suitable to describe these systems, when used in conjunction with our novel dynamical Jastrow factor. We will see that this is not that case for molecules.

The relevant differences can be found, on the other hand, between the binding energies estimated at the VMC level and those predicted by DMC. This is a reasonable result, since the binding that we are measuring is dependent on the strong correlation between the electronic cloud and a single positron. If this electron–positron correlation effect is not exactly described through the trial wave function (which is generally the case also in pure electronic systems), the binding predicted by VMC will be consistently underestimated.

In general though, VMC and DMC agree qualitatively well, except for the cases in which the binding energies are quite small, as for the BE_Ps_ of *e*^+^Li PsLi, PsB, PsC, and the PA of *e*^+^Be, for which in some cases the stability is inverted.

Within this group of systems, *e*^+^Li and *e*^+^Be are two of the most challenging ones, since the rather weak positronic bond is explained by Li and Be atoms’ low ionization potential, low electron affinities, and large covalent radius.^13^ Fortunately, due to the reduced number of Fermions, they have been studied with the most accurate SVM methods^80,81^ that can serve as references. If we compare our accurate DMC (AGP/EPO) results with the SVM ones for the BE_Ps_ energy of *e*^+^Li and the PA energy of *e*^+^Be, we can see that the former are lower of about 0.03 eV with respect to the latter. This is consistent with the corresponding total energies that in our case are compatible with the SP function,^32^ while Bressanini and co-workers have shown the necessity to converge the energy with a MP wave function of up to a linear combination of 111 pairing functions.^32^

Another special case is that of the PsB. This system is stable with respect to the PA dissociation channel, but unstable against the BE_Ps_ one. Moreover, as reported in the previous section, due to the size consistency problem of the AGP or of MR approaches in the description of the B atom and its anion, the error in the estimation of the electron affinity can range between 20% and 100% of the total value. As a consequence, this affects the estimation of the BE_Ps_ energy introducing an error of about 0.08 eV for the AGP wave function at the DMC level (see Table S3 of the Supporting Information). For this reason, we want to focus our attention to boron recomputing the PA and the BE_Ps_ using also an SD wave function to describe the electronic correlation. In Table 2 we compare the total energies of the SD and AGP wave function, used in conjunction with the PMO and EPO positronic ones at both the VMC and DMC levels, and in Table S4 of the Supporting Information we report the numerical values of the PA and BE_Ps_.

As expected, the PA are practically the same between all the wave functions ansätze with VMC and DMC, since there is no change in the number of electrons between the atomic species in eq 19. On the contrary, the positronium dissociation channel involves the removal of one electron, which can be expressed in terms of the EA of the neutral atom as
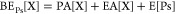
21

For this reason with the SD wave function the BE_Ps_ value is about 200 meV higher with respect to the AGP with both VMC and DMC. The BE_Ps_ DMC energy obtained with the SD/EPO wave function is of −0.4080(32) eV which is only 0.05 eV lower than the predicted FCI extrapolation value and more compatible with respect to the previous DMC estimations.^39^ Qualitatively, we must point out that also with the SD wave function PsB is still unstable with respect to the BE_Ps_ dissociation channel.

In light of these results, we can suppose that a similar effect is behind the underestimation of the binding energy observed for the same BE_Ps_ channel in PsC, where our best DMC value with AGP/EPO is 192 meV away from extrapolated FCI.^79^ In fact, if we take a look at the electron affinity of carbon (reported in Table S3 of the Supporting Information) we can see that the error is about 200 meV for VMC and 100 meV for DMC, which explains the discrepancy with the BE_Ps_ value predicted by the extrapolated FCI.

In conclusion, since the positron or positronium affinities are energy differences, we cannot argue that we actually obtain a better estimation of those quantities, nevertheless our DMC values are shown to be in good agreement with the other references obtained with QMC and CI.^23,24,32,79^

### 4.4 Dissociation Channels of the e^+^·H_2_^2–^ Molecule

**Figure 4:**
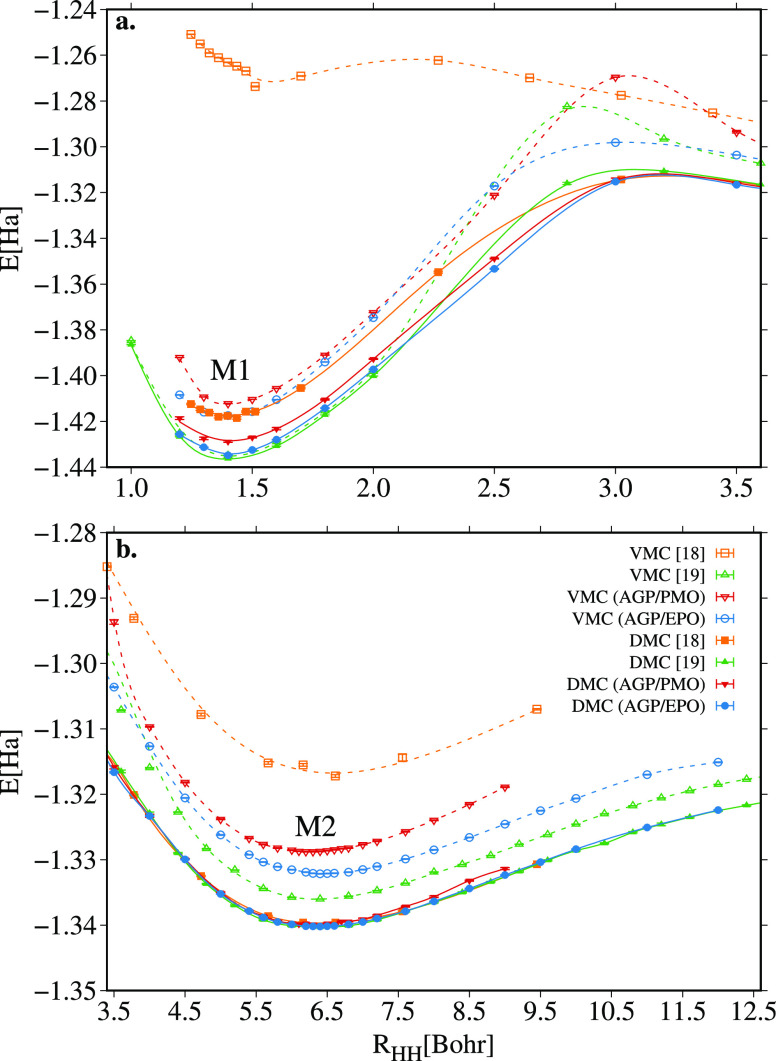
Comparison between the VMC and DMC potential energy surfaces of the e^+^·H_2_^2–^ around the M1 minimum (panel a) and around second minimum M2 (panel b) obtained with the AGP/PMO and AGP/EPO wave functions and with other wave functions presented in the literature.

As a first attempt to study molecular systems, we test the performance of the different wave functions on the dissociation channels of two hydrogen anions bound by one positron:^18,19,61^
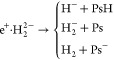
22In Figure 4 we gather all the potential energy surfaces (PES) of the e^+^·H_2_^2–^ molecule as a function of the internuclear distance between the hydrogen atoms, calculated at the VMC and DMC levels with the AGP/PMO and AGP/EPO wave functions, as well as the results previously reported in the literature (see also Table S5 of the Supporting Information). As discussed by Ito et al.^18^ and Bressanini^19^ the PES of the e^+^·H_2_^2–^ molecule has two minima: the first minimum appears at an internuclear distance equal to the equilibrium distance of the two H atoms in the H_2_ molecule, which we referred as M1 in panel a of Figure 4; the second minimum, which we define as M2 in panel b of Figure 4, is observed at larger distances between 5.5 and 6.5 Bohr and is found to be stable with respect to the dissociation of e^+^·H_2_^2–^ in PsH and H^–^, yet, its total energy is higher than the M1 minimum.

From Figure 4, it is clear that at the VMC level the qualitative description of the PES strongly depends on the variational ansatz. The least accurate representation of the molecular dissociation is given by the wave function used by Ito et al.,^18^ which is composed of the product between a Slater determinant (for the electronic wave function), a PMO orbital for the positron, both optimized at the Hartree–Fock level, and a two body Jastrow factor that recovers correlation between electron–electron, electron–nuclei, and electron–positron pairs. Clearly, the two-body Jastrow factor, which is a function that tends to one as the interparticle distances increase, is not suitable to describe the long-range attraction between positrons and electrons, that form bound states. On the other hand, the most accurate wave function is the correlated SP wave function used by Bressanini,^19^ which explicitly includes two particle correlation effects and has been used also for the PsH and PsLi and *e*^+^Li atomic systems described in the section above.

If we focus on the M2 minimum in Figure 4b we can see that our AGP/PMO wave function is better with respect to Ito’s description, due to the combination of the full relaxation of the variational parameters within the VMC framework, and the use of the dynamical Jastrow factor that we introduce in eq 18. The AGP/EPO wave function on the other hand gives results that are more accurate with respect to those of the AGP/PMO, but does not match the accuracy of Bressanini’s SP wave function, differently for what was obtained for the atomic systems. Moreover, the estimated M2 energy minimum of the AGP/EPO wave function is more accurate with respect to that predicted by the AGP/MPO wave function which is slightly shifted toward shorter distances by 0.2 Bohr.

Interestingly, all DMC curves are practically equal for the M2 region, suggesting that the nodal surface is correctly described by all the trial wave functions.

**Figure 5:**
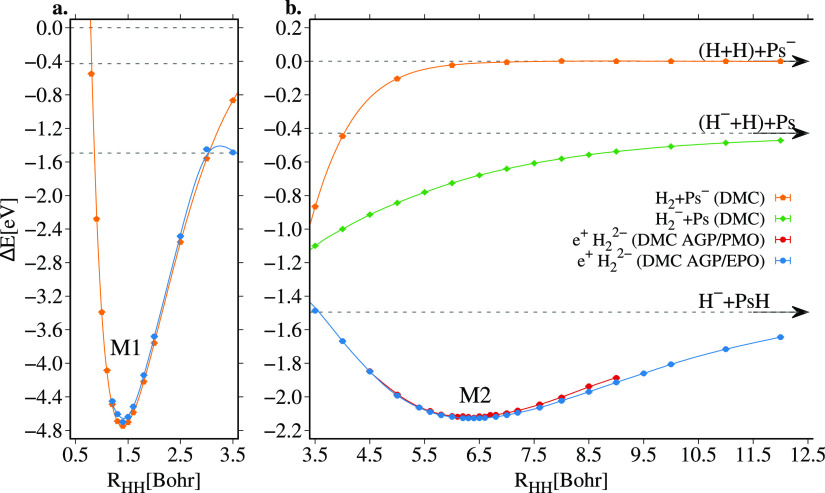
Potential energy surfaces around the M1 minimum of H_2_+Ps^–^ (panel a) and around the M2 minimum of e^+^·H_2_^2–^ (panel b) obtained at the DMC level for the chemical systems consisting of two hydrogen atoms (H) plus the positronium anion (Ps^–^). The dissociation energy of this system in H + H + Ps^–^ fragments, equal to −1.262 Ha, is assumed to be the reference. In orange we show the potential energy curve of the H_2_ molecule shifted by the energy of Ps^–^ (−0.262 Ha). The potential energy surface of the e^+^·H_2_^2–^ molecule is shown for the AGP/EPO (blue circles) and for the AGP/PMO (full red circles). In green we report the potential energy surface of the H_2_^–^ anion shifted by the energy of Ps (−0.25 Ha).

The results around the M1 minimum require further discussion. Bressanini^19^ demonstrated that the M1 minimum is actually the noninteracting state between the H_2_ molecule and the Ps^–^ anion. To describe this region at the variational level, it is essential to have a wave function that can correctly factorize as the product of the two noninteracting subspaces. This is in fact the type of wave function that Bressanini uses to describe this state in ref (19). This is clearly not the case for Ito’s wave function, and for our proposed AGP/PMO. Consequently, taking into account this fragmentation, it is clear why in this case the EPO ansatz is again superior than PMO, since the latter forces the positron to localize around the nuclei, while the EPO gives enough flexibility for the positron to adapt to the distribution of the electronic cloud. As a matter of fact, the relaxation of the AGP/EPO can qualitatively describe this region of space and is remarkably close to the exact H_2_ PES rescaled by the energy of the Ps^–^ anion; yet, also this function cannot fully factorize to a product of the noninteracting subsystems, and this explains the slight error that we have at the VMC level, that disappears when using the higher DMC level of calculation (see also Figure 5).

Following the stability analysis proposed by Bressanini,^19^ in Figure 5 we plot the PES at the DMC level for the e^+^·H_2_^2–^ system as a function of the internuclear distance between the hydrogen atoms. In addition, we also included the PES of H_2_ shifted by the energy of Ps^–^ (−0.262 Ha) as well as the PES of H_2_^–^ shifted by the energy of Ps (−0.250 Ha) (see also Table S6 of the Supporting Information), that allow us to discuss the vertical dissociation channels at each distance. In panel a of Figure 5 it can be seen that around 3.5 bohrs the PES of H_2_ + Ps^–^ and of the e^+^·H_2_^2–^ positronic molecule intersect, thus e^+^·H_2_^2–^ spontaneously dissociates in the H_2_ + Ps^–^ fragments.

This second M2 minimum of the e^+^·H_2_^2–^ molecule, shown in panel b, observed at larger distances, around 6.0 to 6.5 Bohr, is found to be stable with respect to the dissociation in PsH and H^–^ from which it is separated by a potential barrier of 24 mHa, and also against the vertical dissociations in H_2_ + Ps^–^ or H_2_^–^ + Ps. For this minimum we calculate the vibrational parameters using perturbation theory^82^ as also explained in ref (83), through which we obtained an equilibrium geometry of *R*_HH_ = 6.367(5) Bohr, a dissociation energy with and without zero point energy corrections (ZPE) equal to *D*_0_ = 22.31(1) mHa and *D*_*e*_ = 23.35(1) mHa, respectively, ZPE corrections of 229(2) cm^–1^, harmonic vibrational frequency of ω_*e*_ = 461(3) cm^–1^, and a first anharmonicity constant of *x*_*e*_ω_*e*_ = 6(1) cm^–1^. These are the most detailed results, reported up until now in the literature for the M2 minimum of the e^+^·H_2_^2–^ positronic molecule, and our dissociation energy without ZPE only slightly differs from the previous accurate predictions of Bressanini,^19^ of −0.2 mHa, and from those of Ito et al.,^18^ by about 0.6 mHa.

The nature of this particular bond and of the positronic bonds in general is still largely debated. In the literature, the M2 minimum is referred to as a positronic covalent bond,^17,61^ since similarities are observed by comparing, for example, the covalently bound Li_2_^+^ cation (or e^–^Li_2_^2+^) with the corresponding e^+^Li_2_^2–^ positronic molecule. The two molecules were seen to share properties such as equilibrium distances, vibrational frequencies, and binding energies, as well as similarities in the distributions of the electronic HOMO (for e^–^Li_2_^2+^) and of the positron (for e^+^Li_2_^2–^) densities. Later, Goli and Shahbazian,^17^ using AIM analysis, confirmed that the dominant contribution to the bonding is the positron density in between the atoms, which acts as a mediator between the otherwise repulsive anions that do not share any electrons. Furthermore, the authors confirmed a recent study by Nascimento and co-workers^84^ which demonstrated that there was no distinction between the mechanism responsible for the formation of one- and two-electron bonds. Despite this, for systems sharing positron pairs, such as the (PsH)_2_ molecule studied in a recent work by Bressanini,^20^ the author highlights relevant differences with the corresponding electronic bond formation in H_2_ stating that “It remains to be seen if the binding mechanism in (PsH)_2_ is the same as in the H_2_ covalent bond or it is a completely different and new type of bond”.^20^ Moreover, we must add that the bond in the e^+^·H_2_^2–^ molecule shows intriguing similarities with a van der Waals minimum, such as the slow decay of the interaction energy, where two noncovalently bound atoms are energetically stabilized by a delicate balance between the Pauli repulsion and the attractive dispersion effects. For this reason, in the future it will be interesting to study in detail the electronic properties and their response to an external perturbation, which will shed a more complete light on the bonding nature of positronic molecules.

For now, regarding the results presented in this section, we can say that although at the DMC level both the AGP/EPO and AGP/PMO ansätze agree in the description of the M2 minimum, it is evident that the AGP/PMO is overall less accurate. In fact, around the M1 minimum the AGP/PMO is not able to correctly reproduce the nodal surface of the partitioned H_2_ + Ps^–^ system, which on the other hand is better described by the AGP/EPO wave function. Moreover, the AGP/EPO is also able to give a good qualitative description of the system at the level of VMC, due to the more efficient description of the electron–positron correlation effects, also enhanced by the novel dynamical Jastrow factor, which could be suitable to describe loosely Ps or Ps^–^ bound states as well as more localized positronic molecular systems. Finally, at the DMC level the AGP/EPO PES of the e^+^·H_2_^2–^ molecule has been described with a similar accuracy obtained by Bressanini^19^ using DMC applied to an explicitly correlated ansätz. Thus, our accurate approach allows us to provide the most detailed information regarding the vibrational properties of the M2 minimum.

## 5 Conclusions

In the past decade, experimental evidence has accumulated for the formation of metastable states between molecules and positrons, raising interest in the understanding of the binding mechanisms between positrons on the one hand and atoms and molecules on the other. However, the description of such metastable antimatter/matter states represents a difficult challenge for quantum-chemical methods due to the need to describe the strong attractive correlation effects between the electronic cloud and the positrons. The main challenge for quantum chemistry methods lies essentially in the employment of atom-centered basis sets to describe the positronic orbitals. Since the positron does not form bound states with the nuclei, the natural basis would be expanding in terms of Ps orbitals, which explicitly capture the correlation between electron–positron pairs. In this respect, QMC methods have a strong advantage since they are able to incorporate sophisticated wave functions with the ability to explicitly include the correlation effects between particles. Naturally, even in QMC one needs to balance between simplicity and accuracy of wave functions that can be extended to treat large molecular systems without introducing prohibitive computational cost.

In this work we have presented a correlated wave function to study the interaction of a positron with complex atomic and molecular systems. The wave function is constructed as a product of an electronic determinant, in this case the AGP or the SD, a positronic orbital, built of electron–positron correlation function (EPO), and a novel explicit Jastrow factor that includes the correlation between electron–positron pairs in the field of the nuclei. We have compared this approach with the most commonly used methods in quantum chemistry, to study the binding energies of the positron with different atomic systems and with simple molecules for which accurate results have been obtained in the literature.

For atomic systems, the comparison between our two wave functions demonstrates the accuracy of the Jastrow factor and its important role in the recovery of the necessary correlation to obtain an excellent estimation of the binding energies at the level of VMC. The EPO and PMO results are in fact comparable at the VMC level and identical when doing DMC calculations. This agreement between VMC and DMC is explained by the isotropy of the positron wave function, which is centered around the electronic charge and thus can be represented correctly by a basis set composed of atom-centered orbitals.

Importantly, for the heaviest atoms, such as B, C, O, and F, the total energies of the positronic systems PsB, PsC, PsO, and PsF presented in this work are the most accurate in the literature so far and will serve as references for future investigations. For the lighter atoms, such as H, Li, and Be, we have shown that our approaches are comparable to the SP wave functions used by Bressanini in ref (32), yet easier to generalize also to heavier atoms and more complex molecules. Regarding the positron and positronium affinities, the accuracy analysis is not straightforward since these quantities are energy differences and the variational principle cannot be used as a guide; however, our DMC values are in excellent agreement to other references present in the literature.

Clearly, for molecular systems the discrepancy between the EPO and MPO approaches becomes more evident. As a matter of fact, at the VMC level the dissociation curves of the *e*^+^(H^–^)_2_ molecular system, computed with the EPO and PMO wave functions, show a discrepancy in energies of about 0.005 Ha in favor of the former, and more importantly a different structural M2 minimum. Through the EPO wave function, using a dense grid of points, we were able to reconstruct the PES of the M2 minimum and report accurate spectroscopic properties for the *e*^+^(H^–^)_2_ molecule.

In summary, the wave function ansatz proposed in this work has been shown to be efficient in reaching a quantitative description of the binding properties for positronic atoms and molecules with rather small basis sets and lower computational effort with respect to the converged limits of traditional quantum-chemistry approaches. Moreover, the presented wave function is general enough to be applied to larger molecular compounds, taking advantage of the scalability of the QMC methods, paving the way to a systematic and computationally feasible study of large positronic molecular compounds. Furthermore, the EPO wave function will serve as the basis for the implementations of more complex representations, which are necessary to describe systems of many-electrons and many-positrons, such as the extension of the Pfaffian wave function.^85^ For these reasons, our method can be applied in the study of binding mechanisms for a variety of positronic systems, including also the analysis of their electronic and response properties, which are fundamental to shed light onto the physics of these metastable chemical compounds, and to stimulate further experimental studies.

## Supporting Information Available

VMC and DMC energies obtained for the atoms and the atomic ions; values of the electron affinities and ionization potentials corresponding to the atoms studied in this work; values of the positron and positronium binding energies shown in Figures 2 and 3 in the article; potential energy surfaces of the *e*^+^(H^–^)_2_ positronic molecule computed with the PMO and EPO wave functions; potential energy surfaces of the H_2_ molecule and of its anion (PDF)
