# Supplementary material for: Correlated Wave Functions for Electron–Positron Interactions in Atoms and Molecules
Source: J Chem Theory Comput. 2022 Mar 25;18(4):2267–80. doi: 10.1021/acs.jctc.1c01193 (PMC9009097; doi:10.1021/acs.jctc.1c01193)
Supplement: Supplementary file 1 — ct1c01193_si_001.pdf [file ct1c01193_si_001.pdf]

Supporting Information: "Correlated wave functions for electron–positron interactions in atoms and molecules"

Jorge Alfonso Charry Martinez*, Matteo Barborini*, Alexandre Tkatchenko*

*Department of Physics and Materials Science, University of Luxembourg, L-1511 Luxembourg City, Luxembourg

Table S1: Non-relativistic total energies of the atoms obtained with Single determinant (SD) and multideterminantal (MD) wave functions. In parenthesis we report the electronic state of the system. All energies are reported in Hartree.

	Li(^{2}S)	Be(^{1}S)	B(^{2}P)	C(^{3}P)	O(^{3}P)	F(^{2}P)
VMC SD [1]	-7.47683(3)	-14.6311(1)	-24.6056(2)	-37.8147(1)	-75.0233(3)	-99.6874(3)
VMC SD [2]	-7.47693(3)	-14.64622(1)	-24.62361(9)	-37.8083(1)	-75.0229(2)	-99.6877(3)
VMC MD [2]		-14.66480(3)	-24.64432(8)	-37.82972(8)		
VMC MD [1]	-7.47752(3)	-14.66630(4)	-24.65055(6)	-37.8383(1)	-75.0429(2)	-99.7054(3)
VMC AGP[a]	-7.477478(26)	-14.66624(11)	-24.64287(19)	-37.82430(27)	-75.02917(48)	-99.69212(24)
VMC SD[a]			-24.62860(10)			
DMC SD [1]	-7.478 02(1)	-14.65717(7)	-24.63978(5)	-37.8295(1)	-75.0516(5)	-99.7167(8)
DMC MD [1]	-7.478 00(1)	-14.66729(1)	-24.65325(5)	-37.84317(7)	-75.0578(3)	-99.7237(3)
DMC AGP[a]	-7.47805252(60)	-14.667315(90)	-24.65034(19)	-37.83713(22)	-75.0529960(65)	-99.719018(95)
DMC SD[a]			-24.64062(24)			
HF [3]	-7.432726931	-14.57302317	-24.52906073	-37.68861896	-74.80939847	-99.40934939
Exact [4]	-7.47806	-14.66736	-24.65391	-37.8450	-75.0673	-99.7338

[a]This work.

Table S2: Non-relativistic total energies of the atomic ions. All energies are reported in Hartree.

	H^-(^{1}S)	Li^-(^{1}S)	B^-(^{3}S)	C^-(^{4}S)	O^-(^{2}P)	F^-(^{1}S)	Li^+(^{1}S)	Be^+(^{2}S)
VMC SD [5]		-7.49145(6)	-24.63235(6)	-37.85566(8)	-75.0707(1)	-99.8121(2)	-7.279880(10)	-14.32241(18)
VMC MD [5]		-7.49909(2)	-24.6435(3)	-37.8657(1)				
VMC AGP[b]	-0.5275729(51)	-7.49916(36)	-24.644739(85)	-37.86381(11)	-75.08019(18)	-99.81738(22)	-7.279624(27)	-14.324014(35)
VMC SD[b]			-24.63863(12)					
DMC SD [5]		-7.498 58(5)	-24.65230(5)	-37.8788(1)	-75.1027(2)	-99.8434(2)	-7.2799100(50)	-14.3246360(40)
DMC MD [5]		-7.50077(4)	-24.6574(1)	-37.88351(4)				
DMC AGP[b]	-0.527741(23)	-7.50072(19)	-24.65734(26)	-37.88037(40)	-75.10639(19)	-99.84561(66)	-7.279905(32)	-14.324723(31)
DMC SD[b]			-24.65317(19)					
HF [3]		-7.42823206	-24.5192214	-37.7088436	-74.7897459	-99.4594539	-7.23641520	-14.2773948
Exact [4]	-0.527751[a]	-7.50077	-24.6642	-37.8910	-75.1210	-99.8588	-7.27992	-14.32476

[a]SVM calculation from ref. 6. [b]This work.

Table S3: Electron affinities (EA) and Ionization potentials (IP). All values are in eV.

	Electron affinities [eV]						Ionization potentials [eV]	
	H	Li	B	C	O	F	Li	Be
VMC SD [5]		0.395(2)	0.238(3)	1.289(4)	1.301(6)	3.385(9)	5.362(1)	8.812(5)
VMC MD [5]		0.603(1)	-0.022(8)	0.979(3)				9.317(5)
VMC SD[a]			0.273(4)					
VMC AGP[a]	0.7503(2)	0.590(1)	0.051(6)	1.075(7)	1.388(15)	3.409(6)	5.384(1)	9.312(3)
DMC SD [5]		0.559(2)	0.340(2)	1.342(6)	1.37(2)	3.445(8)	5.391(1)	9.050(2)
DMC MD [5]		0.619(1)	0.158(3)	1.161(2)				9.320(1)
DMC SD[a]			0.341(8)					
DMC AGP[a]	0.7549(6)	0.617(5)	0.190(9)	1.177(12)	1.453(5)	3.445(18)	5.392(1)	9.322(1)
Exp	0.754195(19) [7]	0.618049(22) [8]	0.279723(25) [9]	1.2621226(11) [10]	1.461112(3) [11]	3.4011895(25) [12]	5.3917149(4) [13]	9.322699(7) [14]

[a]This work.

Table S4: Positron affinity (PA) calculated as PA[X] = E[X] - E[e^+X] and Positronium binding energy BE_{Ps}[X] = E[X^+] + E[Ps] - E[e^+X]. All energies are in eV. For the energies of Hydrogen and Positronium atoms we used the exact values of -0.5 and -0.25 Hartree respectively.

	PA	BE_{Ps}		PA	BE_{Ps}
	H^-			Li	
VMC AGP/PMO[a]	7.0213(10)	0.9687(10)	VMC AGP/PMO[a]	1.2392(32)	-0.1798(32)
VMC AGP/EPO[a]	7.04347(92)	0.99092(91)	VMC AGP/EPO[a]	1.3112(26)	-0.1078(23)
DMC AGP/PMO[a]	7.1094(35)	1.0614(35)	DMC AGP/PMO[a]	1.433(26)	0.022(26)
DMC AGP/EPO[a]	7.11248(62)	1.064485(86)	DMC AGP/EPO[a]	1.4390(63)	0.0280(63)
MRCI [15]	7.110	1.066	Ref. [16]	1.477	
Hylleras [17]		1.0666	SVM [18]		0.0675
	Li^-			Be	
VMC AGP/PMO[a]	6.0896(26)	-0.1232(24)	VMC AGP/PMO[a]	-0.232(90)	2.277(90)
VMC AGP/EPO[a]	6.1160(26)	-0.0968(24)	VMC AGP/EPO[a]	-0.0646(58)	2.4449(50)
DMC AGP/PMO[a]	6.4613(69)	0.2753(44)	DMC AGP/PMO[a]	0.0340(81)	2.5536(78)
DMC AGP/EPO[a]	6.458(12)	0.272(11)	DMC AGP/EPO[a]	0.054(10)	2.5740(98)
VMC [19]	0.460(16)	-6.1171(82)	Ref. [16]	0.086	2.608
DMC [19]	6.506(22)	0.261(22)	SVM [20]	0.086	
DMC [21]		0.3137(16)			
SVM [20]		0.330564			
	B^-			C^-	
VMC AGP/PMO[a]	5.4667(32)	-1.2854(61)	VMC AGP/PMO[a]	5.536(10)	-0.191(12)
VMC AGP/EPO[a]	5.4808(32)	-1.2713(61)	VMC AGP/EPO[a]	5.556(11)	-0.171(13)
DMC AGP/PMO[a]	6.003(24)	-0.609(23)	DMC AGP/PMO[a]	5.890(24)	0.263(22)
DMC AGP/EPO[a]	6.010(12)	-0.603(11)	DMC AGP/EPO[a]	5.921(19)	0.294(17)
VMC SD/PMO[a]	5.48908(46)	-1.04074(42)			
VMC SD/EPO[a]	5.50594(47)	-1.0239(44)			
DMC SD/PMO[a]	6.00605(87)	-0.45546(94)			
DMC SD/EPO[a]	6.0535(23)	-0.4080(32)			
VMC [19]	3.837(54)	-2.667(54)	VMC [19]	4.354(82)	-1.170(82)
DMC [19]	6.014(27)	-0.435(27)	DMC [19]	5.940(16)	0.479(16)
MRCI [22]	6.176	-0.350	MRCI [22]	6.029	0.486
	O^-			F^-	
VMC AGP/PMO[a]	5.449(23)	0.035(26)	VMC AGP/PMO[a]	5.567(19)	2.173(19)
VMC AGP/EPO[a]	5.536(16)	0.122(19)	VMC AGP/EPO[a]	5.647(16)	2.253(16)
DMC AGP/PMO[a]	6.0138(76)	0.6639(55)	DMC AGP/PMO[a]	6.130(22)	2.772(14)
DMC AGP/EPO[a]	6.076(18)	0.726(17)	DMC AGP/EPO[a]	6.224(19)	2.8663(48)
VMC [19]	2.286(82)	-3.157(82)	VMC [19]	5.306(82)	2.122(82)
DMC [19]	5.861(16)	0.433(16)	DMC [19]	6.169(22)	2.838(22)
MRCI [22]	6.150	0.796	MRCI [23]	6.215	2.806

[a]This work.

References

(1) Brown, M. D.; Trail, J. R.; López Ríos, P.; Needs, R. J. Energies of the first row atoms from quantum Monte Carlo. *J. Chem. Phys.* **2007**, *126*, 224110.

(2) Buendía, E.; Gálvez, F. J.; Maldonado, P.; Sarsa, A. Quantum Monte Carlo ground state energies for the atoms Li through Ar. *J. Chem. Phys.* **2009**, *131*, 044115.

(3) Koga, T.; Watanabe, S.; Kanayama, K.; Yasuda, R.; Thakkar, A. J. Improved Roothaan–Hartree–Fock wave functions for atoms and ions with N≤54. *J. Chem. Phys.* **1995**, *103*, 3000–3005.

(4) Chakravorty, S. J.; Gwaltney, S. R.; Davidson, E. R.; Parpia, F. A.; p Fischer, C. F. Ground-state correlation energies for atomic ions with 3 to 18 electrons. *Phys. Rev. A* **1993**, *47*, 3649–3670.

(5) Maldonado, P.; Sarsa, A.; Buendía, E.; Gálvez, F. J. Quantum Monte Carlo ground state energies for the singly charged ions from Li through Ar. *J. Chem. Phys.* **2010**, *133*, 064102.

(6) Ryzhikh, G. G.; Mitroy, J.; Varga, K. The structure of exotic atoms containing positrons and positronium. *J. Phys. B At. Mol. Opt. Phys.* **1998**, *31*, 3965–3996.

(7) Lykke, K. R.; Murray, K. K.; Lineberger, W. C. Threshold photodetachment of H^-. *Phys. Rev. A* **1991**, *43*, 6104–6107.

(8) Haeffler, G.; Hanstorp, D.; Kiyan, I.; Klinkmüller, A. E.; Ljungblad, U.; Pegg, D. J. Electron affinity of Li: A state-selective measurement. *Phys. Rev. A* **1996**, *53*, 4127–4131.

(9) Scheer, M.; Bilodeau, R. C.; Haugen, H. K. Negative Ion of Boron: An Experimental Study of the ^3P Ground State. *Phys. Rev. Lett.* **1998**, *80*, 2562–2565.

(10) Bresteau, D.; Drag, C.; Blondel, C. Isotope shift of the electron affinity of carbon measured by photodetachment microscopy. *Phys. Rev. A* **2016**, *93*, 013414.

(11) Blondel, C.; Delsart, C.; Valli, C.; Yiou, S.; Godefroid, M. R.; Van Eck, S. Electron affinities of ^{16}O, ^{17}O, ^{18}O, the fine structure of $^{16}O^-$, and the hyperfine structure of $^{17}O^-$. *Phys. Rev. A* **2001**, *64*, 052504.

(12) Blondel, C.; Delsart, C.; Goldfarb, F. Electron spectrometry at the μeV level and the electron affinities of Si and F. *J. Phys. B: At. Mol. Opt. Phys.* **2001**, *34*, L281–L288.

(13) Bushaw, B. A.; Nörtershäuser, W.; Drake, G. W. F.; Kluge, H.-J. Ionization energy of ^{6,7}Li determined by triple-resonance laser

Table S5: Potential energy surfaces of the $e^{+}\mathbf{H}_2^{2-}$ molecule as a function of the nuclei of the Hydrogen atoms. The distances are in Bohr while the energies are in Hartree.

	EPO			PMO		
R_{HH}	VMC	DMC ($dt = 0.010$)	DMC ($dt = 0.005$)	VMC	DMC ($dt = 0.010$)	DMC ($dt = 0.005$)
1.2	-1.40839(9)	-1.42498(13)	-1.42559(14)			
1.3	-1.41594(9)	-1.43158(12)	-1.43125(10)			
1.4	-1.41745(9)	-1.43410(12)	-1.43474(15)			
1.5	-1.41571(9)	-1.43232(16)	-1.43245(9)			
1.6	-1.41050(9)	-1.42863(14)	-1.42798(7)			
1.8	-1.39402(9)	-1.41462(22)	-1.41419(10)			
2.0	-1.37462(9)	-1.39752(22)	-1.39724(14)			
2.5	-1.31716(9)	-1.35373(22)	-1.35331(14)			
3.0	-1.29812(5)	-1.31548(16)	-1.31522(14)			
3.5	-1.30362(5)	-1.31641(8)	-1.31662(11)			
4.0	-1.31266(5)	-1.32334(8)	-1.32330(6)			
4.5	-1.32055(5)	-1.33002(5)	-1.32995(5)	-1.31817(6)	-1.32982(10)	-1.32988(10)
5.0	-1.32621(5)	-1.33511(5)	-1.33523(5)	-1.32381(6)	-1.33491(10)	-1.33500(10)
5.4	-1.32923(5)	-1.33780(5)	-1.33785(5)	-1.32673(5)	-1.33778(10)	-1.33781(10)
5.6	-1.33034(5)	-1.33878(5)	-1.33881(5)	-1.32764(5)	-1.33859(10)	-1.33859(10)
5.8	-1.33107(5)	-1.33948(5)	-1.33952(5)	-1.32820(5)	-1.33948(10)	-1.33937(10)
6.0	-1.33152(5)	-1.33988(5)	-1.33987(5)	-1.32852(5)	-1.33973(8)	-1.33970(10)
6.2	-1.33189(5)	-1.34008(4)	-1.34017(5)	-1.32877(5)	-1.33988(7)	-1.33974(10)
6.3	-1.33207(5)	-1.34005(5)	-1.34018(5)	-1.32876(5)	-1.34005(10)	-1.33988(10)
6.4	-1.33214(5)	-1.34010(5)	-1.34020(5)	-1.32871(5)	-1.34000(10)	-1.34005(10)
6.5	-1.33209(5)	-1.34003(4)	-1.34015(5)	-1.32861(5)	-1.33967(10)	-1.33975(10)
6.6	-1.33206(5)	-1.34002(5)	-1.34011(5)	-1.32846(5)	-1.33954(10)	-1.33982(10)
6.8	-1.33189(5)	-1.33984(4)	-1.33987(5)	-1.32820(5)	-1.33933(10)	-1.33943(10)
7.0	-1.33150(5)	-1.33948(4)	-1.33950(5)	-1.32767(5)	-1.33893(10)	-1.33909(10)
7.2	-1.33103(5)	-1.33886(4)	-1.33898(5)	-1.32716(5)	-1.33841(10)	-1.33851(10)
7.6	-1.32989(5)	-1.33777(4)	-1.33786(5)	-1.32570(5)	-1.33740(10)	-1.33714(10)
8.0	-1.32850(5)	-1.33637(5)	-1.33636(5)	-1.32396(5)		-1.33566(10)
8.5	-1.32659(5)	-1.33437(4)	-1.33440(5)	-1.32154(5)		-1.33319(10)
9.0	-1.32455(5)	-1.33229(4)	-1.33233(5)	-1.31889(6)		-1.33136(10)
9.5	-1.32249(5)	-1.33025(4)	-1.33037(5)			
10.0	-1.32063(5)	-1.32837(5)	-1.32838(5)			
11.0	-1.31699(5)	-1.32502(5)	-1.32506(5)			
12.0	-1.31510(5)	-1.32234(6)	-1.32241(5)			

spectroscopy. *Phys. Rev. A* **2007**, *75*, 052503.

(14) Beigang, R.,; Schmidt, D.,; West, P. J., LASER SPECTROSCOPY OF HIGH RYDBERG STATES OF LIGHT ALKALINE-EARTH ELEMENTS : Be AND Mg. *J. Phys. Colloques* **1983**, *44*, C7–229–C7–237.

(15) Saito, S. L. Multireference configuration interaction calculations of some low-lying states of positronium hydride. *J. Chem. Phys.* **2003**, *118*, 1714–1720.

(16) Harabati, C.; Dzuba, V. A.; Flambaum, V. V. Identification of atoms that can bind positrons. *Physical Review A - Atomic, Molecular, and Optical Physics* **2014**, *89*, 022517.

(17) Yan, Z. C.; Ho, Y. K. Ground state and S-wave autodissociating resonant states of positronium hydride. *Phys. Rev. A* **1999**, *59*, 2697–2701.

(18) Mitroy, J. Expectation values of the e+Li system. *Physical Review A - Atomic, Molecular, and Optical Physics* **2004**, *70*, 25–27.

(19) Bressanini, D.; Mella, M.; Morosi, G. Positronium chemistry by quantum Monte Carlo. I. Positronium-first row atom complexes. *J. Chem. Phys.* **1998**, *108*, 4756–4760.

(20) Mitroy, J. Structure of the LiPs and e+ Be systems. *J. At. Mol. Sci.* **2010**, *1*, 275–279.

(21) Mella, M.; Chiesa, S.; Morosi, G. Annihilation rate in positronic systems by quantum Monte Carlo: e+LiH as test case. *J. Chem. Phys.* **2002**, *116*, 2852–2862.

(22) Saito, S. L. Multireference configuration interaction calculations for complexes of positronium and B, C, N, and O atoms. *Theo. Chem. Acc.* **2006**, *115*, 281–286.

(23) Saito, S. L. Multireference configuration interaction calculations for positronium halides. *J. Chem. Phys.* **2005**, *122*.

Table S6: Potential energy surfaces of the H_2 and H_2^- molecules obtained with the AGP wave function.

	H_2				H_2^-		
R_{HH}	VMC	DMC ($dt = 0.010$)	DMC ($dt = 0.005$)	R_{HH}	VMC	DMC ($dt = 0.010$)	DMC ($dt = 0.005$)
0.6002	-0.76988(2)	-0.77004(1)	-0.770036(5)	3.0	-1.05333(3)	-1.05767(4)	-1.05787(4)
0.7002	-0.92217(2)	-0.92230(1)	-0.922287(5)	3.5	-1.04954(3)	-1.05245(2)	-1.05242(2)
0.8002	-1.02010(2)	-1.02026(1)	-1.020242(5)	4.0	-1.04562(3)	-1.04879(2)	-1.04877(2)
0.9002	-1.08362(2)	-1.08379(1)	-1.083772(5)	4.5	-1.04280(3)	-1.04561(2)	-1.04560(2)
1.0002	-1.12445(2)	-1.12465(1)	-1.124635(5)	5.0	-1.04024(3)	-1.04305(2)	-1.04300(2)
1.1002	-1.14991(2)	-1.15015(1)	-1.150128(5)	5.5	-1.03786(3)	-1.04071(2)	-1.04069(2)
1.2002	-1.16476(2)	-1.16500(1)	-1.164995(5)	6.0	-1.03577(3)	-1.03867(2)	-1.03868(2)
1.3002	-1.17213(2)	-1.17240(1)	-1.172389(5)	6.5	-1.03423(3)	-1.03699(2)	-1.03696(2)
1.4002	-1.17423(2)	-1.17453(1)	-1.174505(5)	7.0	-1.03312(3)	-1.03558(2)	-1.03557(2)
1.5002	-1.17258(2)	-1.17290(1)	-1.172877(5)	7.5	-1.03221(3)	-1.03434(2)	-1.03434(2)
1.6002	-1.16836(2)	-1.16861(1)	-1.168593(5)	8.0	-1.03142(3)	-1.03333(2)	-1.03335(2)
1.8002	-1.15481(2)	-1.15509(1)	-1.155086(5)	8.5	-1.03085(3)	-1.03250(2)	-1.03247(2)
2.0002	-1.13783(2)	-1.13816(1)	-1.138143(5)	9.0	-1.03019(3)	-1.03178(2)	-1.03177(2)
2.5002	-1.09364(2)	-1.09396(1)	-1.093931(5)	10.0	-1.02918(3)	-1.03071(2)	-1.03065(2)
3.0002	-1.05677(2)	-1.05736(1)	-1.057348(5)	11.0	-1.02845(3)	-1.02988(2)	-1.02988(2)
3.5002	-1.03106(2)	-1.03189(1)	-1.031847(5)	12.0	-1.02795(3)	-1.02934(2)	-1.02933(2)
4.0002	-1.01597(2)	-1.01643(1)	-1.016427(5)				
5.0002	-1.00348(2)	-1.00383(1)	-1.003823(5)				
6.0002	-1.00047(2)	-1.00089(1)	-1.000873(5)				
7.0002	-0.99998(2)	-1.00025(1)	-1.000216(5)				
8.0000	-0.99980(2)	-0.99994(1)	-0.999955(8)				
9.0000	-0.99992(2)	-1.00001(1)	-1.000008(5)				
10.0000	-0.99991(1)	-1.00001(1)	-1.000011(5)				
11.0000	-0.99991(1)	-1.00000(1)	-1.000008(5)				
12.0000	-0.99990(1)	-1.00001(1)	-1.000000(5)				
